# Wrapping up the bad news – HIV assembly and release

**DOI:** 10.1186/1742-4690-10-5

**Published:** 2013-01-10

**Authors:** Bo Meng, Andrew ML Lever

**Affiliations:** 1 Department of Medicine, University of Cambridge, Addenbrooke’s Hospital, Cambridge, CB2 0QQ, United Kingdom

**Keywords:** HIV, Budding, Assembly, ESCRT, ALIX, NEDD

## Abstract

The late Nobel Laureate Sir Peter Medawar once memorably described viruses as ‘bad news wrapped in protein’. Virus assembly in HIV is a remarkably well coordinated process in which the virus achieves extracellular budding using primarily intracellular budding machinery and also the unusual phenomenon of export from the cell of an RNA. Recruitment of the ESCRT system by HIV is one of the best documented examples of the comprehensive way in which a virus hijacks a normal cellular process. This review is a summary of our current understanding of the budding process of HIV, from genomic RNA capture through budding and on to viral maturation, but centering on the proteins of the ESCRT pathway and highlighting some recent advances in our understanding of the cellular components involved and the complex interplay between the Gag protein and the genomic RNA.

## Review

It is now over 20 years since the first striking pictures of a failure in the terminal budding process of HIV were published [1]. It took over three years for the findings to be confirmed and validated [2], and this marked the beginning of our understanding of the role of the ESCRT (endosomal sorting complex required for transport) system in the budding of HIV and subsequently in other enveloped viruses. Insights gained from HIV have revealed a wealth of details about normal cellular processes involving the ESCRT proteins, including vesicle budding into endosomal compartments called multi-vesicular bodies (MVB) [3] and the later discovery of the involvement of this process in the terminal events of cell division and cell separation [4]. How ESCRT functions in viral budding in HIV is now understood in remarkable detail; however, there are some notable unanswered questions.

Viral assembly is focused around the major structural protein of the virus capsid - Gag - which is a 55 kDa polyprotein comprising four major subdomains – Matrix (MA), Capsid (CA), Nucleocapsid (NC) and p6. Flanking NC are two small ‘spacer’ peptides SP1 (p2) and SP2 (p1). The assembly of capsid like particles in HIV and other retroviruses can be achieved by the viral Gag protein independently of the presence of any cellular factors [5], and indeed conical structures resembling mature cores can also be formed [6,7], although production of spherical particles is optimized by nucleic acid. Authentic ‘extended’ Gag assemblies with dimensions comparable to a wild type viral capsid can be formed in the presence of lipid membranes and nucleic acid [8]. The NC region of Gag is well established as contributing to Gag assembly probably by ‘bridging’ between individual Gag monomers via genomic RNA (gRNA) [9]. Assembly of viable virus is possible using a minority of NC mutated Gag proteins containing intact late domains (see below) complemented by at least a fivefold excess of Gags with intact NC domains containing a late domain mutation [10], again inferring an important role for RNA binding to the NC domain inducing bridging. Despite the intimate interactions between Gag and RNA preceding and following the virus assembly, RNA in general and specifically the gRNA which is captured and packaged specifically by the viral Gag protein appeared until recently [11] to have no identified role in the ESCRT mediated process of viral assembly. The second conundrum is that whereas in MVB generation, sequential linking of ESCRT-0, I, II and III is essential, in HIV budding it is still unclear how ESCRT-I activates ESCRT-III given the apparent lack of requirement of ESCRT-II for successful budding in HIV [12]. This question has, again very recently, been opened up by some striking *in vitro* assembly studies where ESCRT-II does appear to be an integral part of the Gag budding process [13].

This review is a summary of our current understanding of the budding process of HIV, centering on the proteins of the ESCRT pathway. However, although that is probably the most intensively investigated and documented period in the virus export pathway, it is important to put it in the context of the processes that precede and follow it, but also overlap with it, namely trafficking and assembly, and release and maturation.

### Events preceding viral assembly and budding

**Figure 1:**
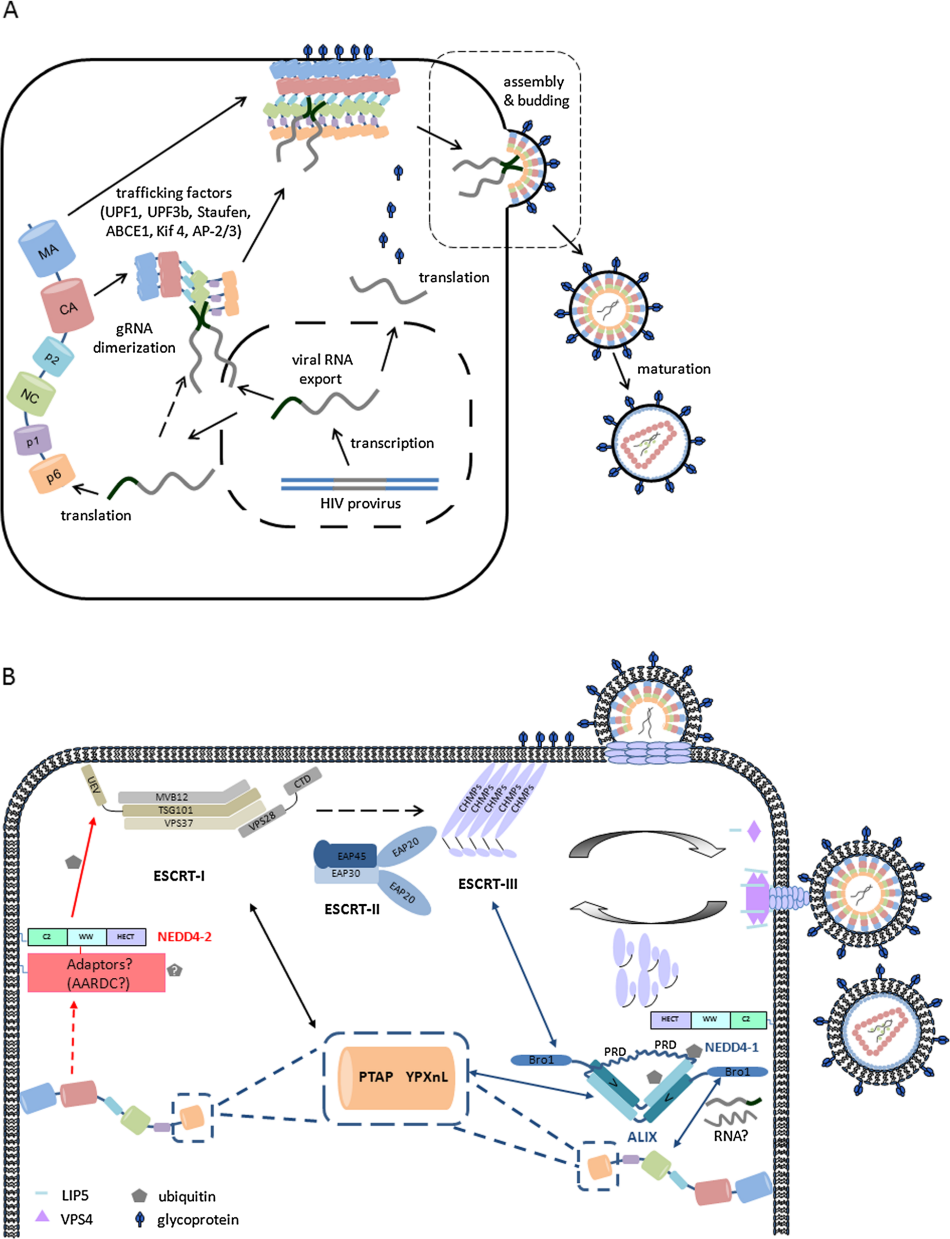
**Diagrammatic overview of the late stages of the HIV life-cycle. (A)** After transcription the full length viral RNA is exported from the nucleus to the cytoplasm for Gag (and Gag-Pol) synthesis. Singly spliced RNA is exported to produce the envelope glycoproteins (dark blue). Gag binds to a dimeric genomic RNA via its NC domain to form ribonucleoprotein complex which is trafficked to the membrane where more Gag assembles and budding occurs (black dashed-box and B). During HIV particle assembly or soon after particle release, protease (PR, not shown) cleaves the Gag polyprotein to MA, CA and NC and protein rearrangement occurs to form the mature virion. **(B)** Close-up view of the HIV budding process. ESCRT-I is recruited to the budding site via interactions between PTAP in p6 of Gag and TSG101 of ESCRT-I (black arrow). The link to ESCRT-III (CHMPs) for budding is via an unknown mechanism but seems to bypass ESCRT-II (black dashed-arrow). Additionally/alternatively, ALIX binds to YPXnL motif also in p6 and NC via V and Bro1 domain, respectively and directs Gag to ESCRT-III via its Bro1 domain for budding (blue arrow). RNA involvement in NC-ALIX binding has been suggested (wavy line), but the identity of the RNA is not clear. HIV has no PPXY motif, but the NEDD4-like ubiquitin ligase family has also been implicated in facilitating HIV joining the ESCRT pathway for budding probably via adaptors on the plasma membrane (NEDD4-2; red arrow) or the ALIX pathway (NEDD4-1). For membrane scission to occur, ESCRT-III components are activated and polymerized to form a dome-shaped structure. VPS4, regulated by LIP5-CHMP5 and CHMP1-ISTI binary complexes (not shown), is recruited to the budding site where it multimerizes to form a dodecamer and disassembles the ESCRT-III filaments for recycling. How membrane scission occurs is unknown.

The earliest cytoplasmic stages of viral nucleocapsid assembly occur at the free cytoplasmic pool of translating ribosomes where unspliced viral RNA is translated to produce Gag (Figure 1A), and a subpopulation of Gag/Pol proteins are generated through a frameshift event. Gag is translated from the RNA species that also functions as the RNA genome. Once translated, the viral gRNA is trafficked away from the translating pool [14]. Although translation may not be a prerequisite for RNA capture [15], subsequent data have reinforced a predominantly cotranslational capture mechanism in HIV-1 [16,17] as shown previously for HIV-2 [18]. The uncleaved Gag polyprotein is involved in RNA genome capture, and the interaction of this with the viral RNA packaging signal [19,20] has been shown in HIV-1 to be biophysically a very different process from that of interaction with the NC sub-fragment of Gag [21]. The latter, however, after cleavage from the precursor protein, is a powerful facilitator of nucleic acid interactions such as those involved in genome dimerization [22,23] and reverse transcription [24-27].

Following capture, evidence suggests that a small number of Gag proteins accompany the gRNA to the budding site at the plasma membrane [28-30]. Currently there is still a relative paucity of knowledge regarding the cellular components of the trafficking Gag/gRNA nucleoprotein complex. UPF1 is suggested to be involved in RNA stabilization [31] in association with UPF3b and Staufen. The cellular ATPase ABCE1 is also implicated in viral assembly in an RNA independent manner [32] and together with DDX6 and other processing body proteins (PBP) facilitate Gag multimerization [33]. The trafficking protein KIF-4 binds Gag [34], but whether this is a requirement for Gag/gRNA complex recruitment at the plasma membrane is unclear. Binding to the clathrin associated adaptor proteins AP-2 and AP-3 has been documented [35,36]; however, the sequential involvement of all these proteins in the Gag/gRNA nucleoprotein complex during its trafficking through the cell is still obscure. In Drosophila the RNA chaperone Staufen and ESCRT proteins interact with RNA [37,38] and influence its directional subcellular trafficking. There is also growing evidence that association of Gag with RNA is important for the directional trafficking of the complex [39]. Staufen has also been implicated in HIV-1 assembly [40,41], but evidence of a close mechanistic link between these factors in HIV-1 assembly is lacking.

Gag binds the viral RNA through interactions involving the NC domain of the polyprotein which itself contributes specificity of RNA selection [42]. RNA also binds non-specifically to a highly basic region of the MA domain reducing non-specific binding to lipid membranes [43] and increasing the specificity of MA for lipid membranes containing phosphatidylinositol (4.5) diphosphate (PIP2) [8] such as the plasma membrane. A saturated lipid myristic acid covalently linked to the N-terminal region of Gag is sequestered in a hydrophobic pocket at the N terminus of MA and can be ejected by pH changes [44], the so called ‘myristyl switch’ mechanism [45]. This conformational change can also be evoked by PIP2 [46]. A model has been proposed whereby Gag bound to RNA through both MA and NC arrives at the plasma membrane where the presence of PIP2 and the effect of Gag multimerization displaces the RNA from the MA region and unleashes the myristic acid to provide a double anchor to the plasma membrane consisting of both myristate and PIP2 [46].

There is an estimated 5000 Gag proteins in the immature virion which are reduced to around 1500 in the mature particle [47], and yet relatively few escort the gRNA to the budding site. Possibly other nucleic acid molecules fulfill the gRNA role in masking the highly basic MA domain and favoring plasma membrane binding over other cellular membranes in the remainder of the Gag monomers that congregate there.

## The viral budding process

### Viral late domains

The essential role of late (L) domains in retrovirus budding first came to attention from the observation of a failure to complete budding seen in deletion and mutation of the terminal component of the HIV-1 Gag polyprotein p6 [1,2]. Two late domains have subsequently been identified within the p6 region of the HIV-1 Gag: the PTAP and YPXnL (where X refers any amino acid and n = 1 ~ 3 residues) motifs (Figure 1B). PTAP was noted to be conserved in HIV-1 and HIV-2 and other retroviruses and was later identified in members of the *Filoviridae*, *Arenaviridae* and *Hepeviridae* families [48,49] . PTAP-TSG101 interaction was originally identified by yeast two hybrid studies [50,51]. Functional analyses of disrupting this interaction by siRNA [50], mutagenesis [52], or *trans* dominant inhibition by TSG101 fragments [53] recapitulates classic phenotype of L-domain-defective HIV.

The YPXnL late domain was first identified in the equivalently sited p9 of EIAV (equine infectious anemia virus) Gag [54] and subsequently in p6 of HIV-1 Gag [55] and p2b of RSV (Rous sarcoma virus) Gag [56]. It promotes budding by binding to ALIX (apoptosis-linked gene 2-interacting protein X), an associated protein of the ESCRT pathway; although the binding affinity of ALIX to the YPXnL motif in HIV-1 is lower than to the corresponding EIAV region [57].

Other retroviruses, including murine leukemia virus (MLV) [58,59], RSV [60-62], Mason-Pfizer monkey virus (M-PMV) [63,64], and Human T-cell leukemia/lymphoma virus type 1 (HTLV-1) [65-67], use a PPXY late domain (where X refers any amino acid) for virus budding. The PPXY late domain was first described in the Gag region of RSV [61] and has since been identified in different virus family members of the *Filoviridae, Arenaviridae* and *Rhabdoviridae*[48]. These motifs bind to the WW domain of NEDD-like HECT ubiquitin ligases [68]. The link between the PPXY late domain and the ESCRT pathway is unknown. However, it has been suggested in HIV that NEDD-like ligase either ubiquitinates and activates ESCRT-I [69], or interacts with ALIX to function in virus release [70]. Central to the function of the late domains are the proteins of the ESCRT pathway.

### ESCRT proteins

The ESCRT system, first elucidated in yeast [71,72], consists of four sets of different cellular proteins (ESCRT-0, I, II and III) that are recruited sequentially to endosomal membranes for biogenesis of MVBs and sorting of the ubiquitinated cargos into those vesicles [73]. Additionally, members of ESCRT or ESCRT-associated proteins are involved in cytokinesis [74], microvesical shedding [75], exosome biogenesis [76] and ubiquitination-independent endosomal sorting [77].

#### ESCRT-0

ESCRT-0 comprises two components STAM (signal transducing adaptor molecule) and HRS (hepatocyte growth factor (HGF)-regulated tyrosine kinase substrate) in humans (Vps27 and Hse1 are the homologues in yeast) and is the least conserved part of the ESCRT pathway. ESCRT-0 is essential for recognition of ubiquitinated cargos at the endosomal membrane and HRS interacts with TSG101 of ESCRT-I to cascade the sorting of cargos and MVB formation. Despite the apparent lack of requirement of HRS for HIV budding [78], a genome-wide screen has identified HRS as a host factor required for HIV infection [79] subsequently confirmed by an siRNA knockdown study which reduced HIV release to less than 10% of wild type (wt) levels [80]. The phenotype is distinct from classic ESCRT related budding defects [50]. Depletion of HRS arrests HIV-1 particles at the cell surface and in endosomes in a similar manner to that seen in the BST-2 expressing cells infected with Vpu-defective HIV-1 [81]. Further analysis has shown that HRS is required for efficient HIV-1 release by facilitating Vpu-induced BST-2 downregulation and degradation [80].

#### ESCRT-I

ESCRT-I includes TSG101 [50,82], VPS28 [83-85], one copy of one of the four versions of VPS37 (A-D) [86-88] and one of the two versions of MVB12 (A&B) [84,89,90] in a stoichiometry of 1:1:1:1 [84,89,91]. The budding of HIV requires the intact ESCRT-I, although the involvement of VPS37A and VPS37D in this process have not been reported. Human ubiquitin associated protein-1 (UBAP-1) is the newly identified member of ESCRT-I [92] involved solely in ubiquitinated endosomal cargo degradation but not in HIV budding or midbody abscission [93,94]. The heterotetrameric complex of ESCRT-I in yeast consists of a globular headpiece with all four components (Vps23, Vps28, Vps37, and Mvb12; orthologs respectively of TSG101, VPS28, VPS37, and MVB12 in humans) attached to an extended stalk composing Vps23, Vps37 and Mvb12 [84].

The human HRS protein from ESCRT-0 recruits TSG101 by binding to its UEV (ubiquitin E2 variant) domain [78]. Hence it was proposed that Gag could mimic this TSG101 recruiting ability by directly interacting with UEV. Indeed structural studies of the UEV domain in complex with the late domain PTAP motif has shown the PTAP peptide binds UEV in a bifurcated groove above the inactive enzymatic site [95]. This was further supported by the demonstration that a PTAP budding defect could be rescued when an HRS TSG101 binding fragment is fused to a PTAP L domain-deleted Gag [78]. Very recently, Nabhan *et al.* proposed that in order to mediate virus budding from host cells HIV Gag (and likely other viral proteins) alternatively have evolved to mimic arrestin domain containing protein-1 (ARRDC1)-mediated ARRDC related microvesicle (ARMMs) release where both TSG101 and VPS4 (AAA ATPase for disassembling the ESCRT-III and see below) are required [75]. ARRDC1 is directed to the plasma membrane by its arrestin-domain and has been previously implicated in functioning as an adaptor in PPXY-dependent budding [96].

Disruption of VPS28 binding to TSG101 causes the arrest of HIV budding suggesting VPS28 plays an integral role in this process [87,97]. The C-terminal domain of VPS28 binds to the ESCRT-II complex [98], and fusion of this region to Gag late domain deletion construct rescues an EIAV budding defect [85]. Additionally, VPS37B and C can rescue a late domain budding defect when fused to the PTAP late domain deficient Gag, consistent with the notion that VPS37 is also part of ESCRT-I and involved in virus budding [87,88]. MVB12 appears to be the exception in that the budding process is not affected when it is depleted [89], but both depletion and overexpression of MVB12 reduce virus infectivity.

#### ESCRT-II

ESCRT-II forms as a ‘Y’ shaped heterotetramer including two copies of Vps25/EAP20, one each of Vps22/EAP30 and Vps36/EAP45 [99]. In yeast, ESCRT-II physically bridges the ESCRT-I and ESCRT-III complexes and is essential for MVB protein sorting and vesicle formation [100-102]. In humans, the interaction between ESCRT-I and ESCRT-II is slightly different due to the lack of an NZF (Npl4-type zinc-finger) motif, which forms the interface in the ESCRT-I/ESCRT-II complex in yeast [101,103]. The interaction between ESCRT-II and ESCRT-III, however, is similar to that in yeast [99,100].

Using purified yeast ESCRT components with giant unilamellar vesicles (GUV), Wollert and Hurley [102] demonstrated that ESCRT-0 clusters ubiquitinated cargo on the membrane, and ESCRT-I together with ESCRT-II deform the membrane where the cargo is encapsulated. Both ESCRT-I and II are found on the outside of the bud where the ESCRT-III is recruited to cleave the bud to form the intralumenal vesicles. However, a role for ESCRT-II in HIV budding was not clear until this same group recently demonstrated that in the GUV system ESCRT-II is indeed recruited and co-localized with ESCRT-I on the Gag assembly site [13]. This is in marked contrast to evidence from siRNA knock down studies *in vivo*[12] which suggest that ESCRT-II is dispensable. This apparent discrepancy is puzzling but may be due to incomplete protein depletion by siRNA if only a small amount of ESCRT-II is needed for HIV-1 budding. Alternatively there may be some unknown additional pathway bridging ESCRT-I and -III *in vivo*.

#### ESCRT-III

The core structure of ESCRT-III includes 12 members of the charged multivesicular protein (CHMP) family (CHMP1A, 1B, 2A, 2B, 3, 4A-C, 5, 6, 7 and IST1). Structural studies of some of the CHMP proteins have shown that they share an N-terminal 4-helix-bundle core structure mediating membrane binding and filament formation [104-106]. The C-terminal tail is adapted as in either auto-inhibition status by folding back on the core or in an activated position for oligomerization [104,107] and also contains MIM (MIT (microtubule-interacting and transport) domain interacting motif) sequence(s) that interacts with MIT-containing proteins in a versatile fashion [108-114]. The involvement of ESCRT-III in retroviral budding was originally suggested from studies of a dominant-negative VPS4 which demonstrated a late domain deficient HIV budding phenotype [50,115]. Later certain CHMP members were also shown to have similar effects when overexpressed as fusion isoforms [55,116,117]. More recently, it was shown that introducing certain mutations in the ALIX/CHMP4 binding site can abolish ALIX-mediated budding [118,119] confirming the integral role of ESCRT-III during the budding process. Triggering of ESCRT-III can potentially be achieved by Vps36/EAP45 binding to CHMP6 [117]; VPS28 binding to CHMP6 [85] or via the associated protein ALIX, which binds to TSG101 and CHMP4 [118,119].

In yeast, Vps20/CHMP6, Snf7/CHMP4, Vps24/CHMP3 and Vps2/CHMP2 are sequentially recruited to the membrane and deform the membrane *in vitro*[120]. Vps 20 plays an important role in bridging with Vps 25 of ESCRT-II and triggering the polymerization of Snf7 [121], although it is not required in HIV budding *in vivo*[12,122]. ALIX may provide an alternative route by linking CHMP4 of ESCRT-III [123]. Alternatively, it is also possible that other undefined host factor(s) exist that fulfil the link between Gag and budding.

Despite the evidence of sequential recruitment of CHMP in yeast, *in vivo* depletion studies in mammalian cells have shown that only CHMP2 & 4 are involved in HIV budding [122], although new findings have suggested CHMP3 also plays a synergistic role with CHMP2 [124]. In contrast to those *in vivo* studies, recent *in vitro* reconstitution in GUV using purified ESCRT complex and HIV Gag has demonstrated that the co-localization of CHMP4 with Gag is maximal only in the presence of CHMP6 and other upstream ESCRT complexes implying the HIV budding route is analogous to that of MVB formation in yeast [13]. Although the exact involvement of some of the individual CHMP proteins is not yet certain, CHMP4 in budding formation is confirmed in both *in vitro* reconstitution [102,125] and *in vivo* studies [122,126].

Once CHMP protein is activated and recruited to the membrane, it is thought to oligomerize. Indeed, several studies have demonstrated that CHMP proteins can form filaments [104,126-129], and it has been hypothesized that the formation of such filament would deform the membrane. This, together with oligomerized and activated VPS4, may ultimately provide sufficient force for scission to occur, although VPS4 independent budding is also documented [102,125]. Interestingly, observation of such polymerized CHMP proteins *in vivo* has largely been unsuccessful apart from overexpression of CHMP4 [126] and CHMP2B [129].

Vta1/LIP5, Did2/CHMP1, Ist1/IST1 and Vps60/CHMP5 regulate and recruit VPS4 to the budding site [107,130-135]. VPS4 exists as a single isoform in yeast but is present as two isoforms in humans (VPS4/SKD1A&B) [136]. It contains an N-terminal MIT domain, a link segment and a single ATPase cassette. MIT domain binds to MIM1 [109,113] and MIM2 [108,110] of subset of CHMP proteins and both MIM1&2 binding sites are required for recruiting VPS4A to endosomal membrane and VPS4B for HIV budding [108]. VPS4 exists as a catalytically inactive dimeric form in the cytoplasm but once recruited to the membrane-associated ESCRT-III, it forms a catalytically active dodecamer [115,137]. ESCRT-associated protein LIP5 is believed to play an important regulatory role in VPS4 oligomerization [138]. It functions by bridging VPS4 through VSL (Vta1/SBP1/LIP5) domain and ESCRT-III by its tandem MIT domains located at the N terminus [111,138,139]. Given the regulatory role of LIP5 in VPS4 oligomerization, it is probably not surprising that depletion of LIP5 also decreased HIV budding [134]. The formation of dodecamer complexes of VPS4 disassemble ESCRT-III through an ATP-driven process which is poorly defined. However, from the nature of VPS4 as an AAA-type ATPase, it has been suggested that ESCRT-III is engaged by passing through the central pole of VPS4 dodecamers [140,141].

Investigation of how membrane scission occurs is still an area of great interest. Several schools of thoughts exist. One model suggests that the depolymerization on ESCRT-III filaments by VPS4 provides enough force to the underlying membrane for scission to occur [120]. It has been shown microscopically that VPS4 is recruited to the membrane abscission site during cytokinesis [142] and retrovirus budding [143,144] before the cleavage occurs supporting the notion that VPS4 may provide the constriction for membrane scission. However, VPS4 independent membrane scission has also been observed *in vitro*[102,125]. Other membrane scission models have also been proposed. Based on the observations that various members of ESCRT-III can form a filamentous structure *in vitro*[104,127,128] and *in vivo*[126,129], it was proposed that the progression of this filament would lead to thinning of the plasma membrane and to a certain degree the energy accumulated would favor fission [145]. Recent studies have suggested that CHMP4 polymerization is regulated by CC2D1A and CC2D1B since when CC2D1A is overexpressed HIV budding is inhibited and conversely if CC2D1 is depleted HIV budding is enhanced [146,147]. A further alternative has recently been proposed suggesting that individual ESCRT-III filaments are sufficient to promote the scission [148]. This model is based on the observation that Vps32/CHMP4 is recruited by ESCRT-II and the Vps20/CHMP6 complex to the curvature region of the membrane and as such modulates the mobility of the membrane. It was proposed that when the binding energy between ESCRT-III filament and membrane exceeds the energy barrier to scission, vesicles would more likely to be pinched off from the membrane.

### ALIX

ALIX is a mammalian homologue of Bro1 in yeast, originally identified as a member of class E VPS class genes from yeast [149]. Subsequently, HIV has also been shown to be able to utilize ALIX to bud from the cells, although this pathway appears to be subordinate to the ESCRT-I route [118,150]. Unlike the classical ESCRT pathway where ESCRT-I and -III in conjunction with a PTAP late domain are involved in virus budding, ALIX-mediated budding relies on the interaction with a different late domain, YPXnL, which is also located within the p6 region of Gag in HIV-1. No YPXnL-domain-containing cellular proteins had been identified *in vivo* until recently when it was shown that ALIX binds to syntenin, functioning in exosome biogenesis [76] and protease-activated receptor 1 (PAR1) for ubiquitination-independent endosomal cargo sorting [77].

ALIX contains an effector V domain for binding to the YPXnL motif [118,151] and polyubiquitin [152]. This is flanked by an N terminus Bro1 domain and a proline-rich C-terminal domain (PRD). The PRD also binds to TSG101, endophilin CIN85 and ALG-2 [153,154]. Although both monomers and dimers were seen [118,155], the active conformation of ALIX is a dimer [123,156,157]. The ALIX pathway was first discovered in studies using a Gag that lacked a functional TSG101-binding site [55,117]. It was shown that, when overexpressed, ALIX rescued the budding defect and this rescue is fully dependent on the YPXnL late domain [118,119] and intact polyubiquitin binding sites on V domain [152]. In addition, structural and biochemical analyses of an ALIX homologue in yeast [158] or ALIX itself [118,119,159] have shown that mutating some residues in the hydrophobic patch of Bro 1 domain abolishes its interaction with CHMP4 and as such eliminates its ability to rescue the budding defect. Interestingly, when overexpressed, Bro 1 rescues the release of HIV-1 lacking both PTAP and YPXnL motifs [160]. Functional analysis has shown that ALIX mediated rescue also requires the NC domain in Gag [160,161]. The interaction between ALIX and the NC domain was documented as being insensitive to benzonase [161], a powerful nuclease. However, very recent data suggest that the interaction between the NC domain of Gag and the Bro1 domain of ALIX is RNA dependent [11], although there was no evidence that it was specific for viral RNA. If the latter is validated it would in part answer the question as to the involvement of RNA in this stage of the assembly process. The C-terminal PRD domain is also essential for rescuing ALIX mediated virus budding, although the binding sites with TSG101, endophilin, CIN85 and CMS are dispensable for the function of ALIX in HIV-1 budding [118,119]. The PRD domain also regulates the ALIX function by folding back against the upper domains and as such auto-inhibits YPXnL late domain binding site on the V domain [157,162,163].

### NEDD4-like ubiquitin ligases

There are several members of the NEDD-like ubiquitin ligase family. They all contain an N terminus C2 domain for membrane binding [164], two to four WW (Trp-Trp) substrate-binding domains and a C-terminal HECT (homologous with the E6-associated protein C-terminus) catalytic domain for targeting ubiquitination. The PPXY late domain was first described in the Gag protein of RSV [60,62], which functions by binding to the WW domain of NEDD-like HECT ubiquitin ligases. Despite the lack of a PPXY late domain in HIV-1, overexpression of NEDD4-1 or 4-2 nevertheless stimulates the PTAP late domain mutant defective budding [69,70]. Interestingly, the most potent effector in rescuing the budding defect is NEDD4-2s/ΔC2 [165], a natural isoform of NEDD4-2 in which most of the C2 domain is truncated [166]. Additionally, neither the PTAP nor the YPXnL domain is required for NEDD4-2s mediated budding [69,165]. To confer a rescue effect, NEDD4-2s must be catalytically active suggesting ubiquitination of viral or cellular proteins is needed [69,165]. The remaining C2 domain is also important in binding to Gag and itself is sufficient to confer HIV-1 budding ability on various members of NEDD family [167]. Depletion of TSG101 abrogates the NEDD4-2s mediated remediation of budding defect, suggesting that ESCRT-I is recruited in this process [69]. Like NEDD4-2, NEDD4-1 can also rescue the PTAP late domain budding defect, albeit less efficiently than NEDD4-2 [69,70]. Additionally, the effect of NEDD4-1 on budding functions in a different manner that is independent of cellular TSG101 and requires the YPXnL late domain. This implies that ALIX recruits NEDD4-1 to mediate HIV budding through the YPXnL-ALIX budding pathway [70].

### Post budding Gag processing

Formation of the budded particle and final scission from the cell is associated with the processes of proteolytic processing of the Gag polyprotein into its component fragments, morphogenesis of the spherical particle into the MA lined enveloped virion with the conical core and maturation of the loosely associated paired RNA genomes into a mature tightly linked dimer. Genomic RNA has been suggested in the past to have a structural scaffolding role aiding viral assembly [6,168,169] and even trace amounts of nucleic acid appear to enhance *in vitro* assembly. Gag dimerization has been implicated as an important first step [170,171]. Mutations that disrupt the dimer interface between the CA domains prevent correct assembly [172]; a plausible role for the gRNA is that it also helps to bridge Gag dimers [173]. The MA and CA domains of Gag foster formation of hexameric arrays of the polyprotein but during cleavage the N-terminal domain of CA can form both pentamers and hexamers and it is this that generates the steric capability to form the asymmetric fullerene cone of the mature capsid [7,174-176].

Release of the homodimeric aspartyl protease (PRO) from Gag/Pol is initiated in an as yet obscure manner although there is evidence for the involvement of clathrin [177,178], but once this has begun autocatalytic cleavage of the remaining Gag and Gag/Pol proteins can occur. Proteolytic cleavage of the Gag and Gag/Pol polyproteins by the viral protease is associated with an alteration in the nature of the dimer interaction from rather loosely associated molecules to a tight dimer. It has become apparent that all these processes are closely interconnected [179-181]. In HIV-2, it has been shown that the proteolytic cleavage of Gag and the packaging of dimeric RNA are intimately inter-dependent [180]; failure of RNA dimerization leads to aberrant proteolytic cleavage of Gag. The reason for this is not clear but it may be due to the lack of an RNA scaffold in bringing together two protease monomers within two Gag/Pol polyproteins and facilitating cleavage at the correct rate and site. Conversely, it has been known for a considerable time that a protease deficient HIV produces poorly dimeric RNA genomes [23] and the mis-processing of HIV-2 Gag was shown to correct in long-term culture by reversion mutation in the MA portion of Gag and this unexpectedly, despite the persistence of the RNA dimer mutation, led to a return of packaging wt levels of dimeric RNA [180]. Similar reversion mutations in the HIV-1 Gag have been seen which restore replication to dimer mutants [182] although the analysis did not reveal whether the RNA dimerization mutation had been left unchanged or RNA dimer packaging had been restored.

## Conclusions

Despite remarkable progress in understanding this intimate interaction between the virus and the cell in which results in viral egress, the late stage of the viral life cycle is still a patchwork of areas of better and less well understood steps. At present it resembles a jigsaw puzzle with missing pieces. The central process of ESCRT mediated budding is fairly well established, but there are still controversies; and the significance of the recently identified variants of some of the ESCRT components is still unclear. The roles of gRNA, in assembling what is primarily a nucleoprotein complex, are only just beginning to be understood. There are gaps in our understanding of the connection of gRNA capture to budding although interdependencies between RNA and protein processing are beginning to emerge. Understanding the assembly process is critically important not least because the diverse interactions with cellular components are promising drug targets since the scope for viral mutational escape is limited due to the unchanging nature of the essential cellular factors involved. More work is needed to fill in the missing links in this remarkable process and to identify susceptible areas for therapeutic intervention.

## Abbreviations

HIV: Human immunodeficiency virus; EIAV: Equine infectious anemia virus; MLV: Murine leukemia virus; RSV: Rous sarcoma virus; M-PMV: Mason-Pfizer monkey virus; HTLV-1: Human T-cell leukemia/lymphoma virus type 1; ESCRT: Endosomal sorting complex required for transport; MVB: Multivesicular bodies; TSG101: Tumour susceptibility gene 101; CHMP: Charged multivesicular body proteins; GUV: Giant unilamellar vesicles; NEDD: Neural precursor cell expressed developmentally down-regulated protein; HECT: Homologous with E6-associated protein C-terminus; VPS: Vacuolar protein sorting; ALIX: Apoptosis-linked gene 2-interacting protein X; HGS: Hepatocyte growth factor-regulated tyrosine kinase substrate; STAM: Signal transducing adaptor molecule; MIT: Microtubule-interacting and transport molecules; MIM: MIT domain interacting motifs; ARRDC 1: Arrestin domain-containing protein 1; ARMM: ARRDC1-mediated microvesicles; HRS: hepatocyte growth factor (HGF)-regulated tyrkinase substrate; NZF: Npl4-type zinc-finger; IST-1: Increased sodium tolerance-1; CIN85: Cbl-interacting protein of 85 kDa; EAP: ELL-associated proteins.

## Competing interests

The authors declare they have no competing interests.

## Authors’ contributions

BM and AL both wrote the manuscript. Both authors read and approved the final manuscript.
